# IMP3 signatures of fallopian tube: a risk for pelvic serous cancers

**DOI:** 10.1186/s13045-014-0049-5

**Published:** 2014-07-12

**Authors:** Yiying Wang, Yue Wang, Dake Li, Lingmin Li, Wenjing Zhang, Guang Yao, Zhong Jiang, Wenxin Zheng

**Affiliations:** 1 Department of Obstetrics and Gynecology, Henan Provincial People’s Hospital, Zhengzhou, China; 2 Department of Pathology, University of Arizona College of Medicine, Tucson, AZ, USA; 3 Department of Obstetrics and Gynecology, Jiangsu Provincial Hospital of Traditional Chinese Medicine, Nanjing, Jiangsu, China; 4 Department of Pathology, Shanxi Medical University, Shanxi, China; 5 Department of Obstetrics and Gynecology, Qilu Hospital, Shandong University, Jinan, Shandong, China; 6 Arizona Cancer Center, University of Arizona, Tucson, AZ, USA; 7 Department of Molecular & Cellular Biology, University of Arizona, Tucson, AZ, USA; 8 Department of Obstetrics and Gynecology, University of Arizona, Tucson, AZ, USA; 9 Department of Pathology, University of Massachusetts Medical Center, Worcester, MA, USA

**Keywords:** IMP3 signature, Fallopian tube, Tubal secretory cells, Ovarian cancer, Pelvic serous carcinoma

## Abstract

**Background:**

Recent advances suggest fallopian tube as the main cellular source for women’s pelvic serous carcinoma (PSC). In addition to *TP53* mutations, many other genetic changes are involved in pelvic serous carcinogenesis. IMP3 is an oncofetal protein which has recently been observed to be overexpressed in benign-looking tubal epithelia. Such findings prompted us to examine the relationship between IMP3 over-expression, patient age and the likelihood of development of PSC.

**Methods:**

Fallopian tubes from three groups (low-risk, high-risk, and PSC) of patients with matched ages were studied. Age was recorded in 10 years intervals ranging from age 20 to older than 80. The number of IMP3 signatures (defined by 10 or more tubal secretory cells stained positively and continuously in benign appearing tubal mucosa) from both tubal fimbria and ampulla segments was measured. The data was analyzed by standard contingency table and Poisson distribution methods after age adjustment. IMP3 overexpression was also examined in serous tubal intraepithelial carcinoma and PSC.

**Results:**

The positive IMP3-stained cells are mainly tubal secretory cells. The absolute number of tubal IMP3 signatures increased significantly within each age group. Age remained a significant risk factor for serous neoplasia after age adjustment. IMP3 signatures were more frequent in the patients of both high-risk and PSC groups. The presence of IMP3 signatures in tubal mucosa was significantly associated with tubal or pelvic serous carcinogenesis (*p* < 0.001).

**Conclusions:**

The findings suggest that tubal secretory cells with IMP3 signatures showing growth advantage could potentially serve as a latent precancer biomarker for tubal or pelvic serous carcinomas in women.

## Findings

The number of tubal IMP3 signatures increased with age, which served as a significant risk factor for serous neoplasia. Identification of IMP3 signatures in tubal mucosa was associated with tubal or pelvic serous carcinogenesis (p < 0.001).

### Introduction

Pelvic serous carcinoma (PSC), including serous cancers of the ovary, peritoneum, and fallopian tube, is the most common and lethal type of mullerian malignancy, comprising more than 70% of all malignancies from these organs [[1]–[3]]. Prognosis of PSC is typically poor since the majority cases present clinically in advanced stages. Therefore, investigators have emphasized the importance of understanding early phases of this disease, including precancer and latent precancer conditions [[4]–[6]]. Important advances in recent years suggest that precancerous lesions of PSC originate from the fallopian tube, rather than the ovary or peritoneal surface [[2],[7]–[12]]. Within the fallopian tubal mucosa, there are two different cell types, ciliated and non-ciliated cells. The latter are also called secretory cells. It is the secretory cell which serves as the cell of origin for the majority of PSC [[2],[7],[8],[13]–[15]]. A proposed model for pelvic serous carcinogenesis starts with an increased secretory to ciliated cell ratio within the fallopian tube epithelia and subsequent loss of *TP53* function and emergence of tubal epithelial “p53 signature”, which is considered as a latent precancer associated with clonal alterations of p53 expression [[15]–[17]]. This latent precancer shares attributes with women’s PSC and has been demonstrated to exist in anatomic continuity with serous tubal intraepithelial carcinoma (STIC), the earliest morphologically identified form of serous carcinoma within the pelvis [[18]–[21]].

With confidence that tubal secretory cells are the origin of pelvic HGSC, many investigators have focused their attention on this particular group of cells within the tubal epithelia. Tubal secretory cell outgrowths (SCOUTs), defined at the cellular level as non-interrupted growth of at least 30 secretory cells, was the first term defined by Crum et al. [[13]]. More recently, we have re-examined the issue and found secretory cell expansion (SCE), defined as ≥ 10 secretory cells in a row, is a more sensitive biomarker than SCOUTs for pelvic serous carcinogenesis [[15]]. This was supported by the observation that SCEs were found in a significantly higher frequency in tubal mucosa from patients with high-risk of development of PSC [[15]]. Morphologically, SCEs are visible under light microscope and, therefore, may be used as a reliable surrogate biomarker for PSC screening and potential cancer prevention. However, the molecular mechanisms leading the SCEs to the development of PSC remain largely unclear.

IMP3, an oncofetal protein, is a member of insulin-like growth factor II mRNA binding proteins, also known as IGF2BP3 [[22],[23]]. IMP3 is epigenetically silenced soon after birth, with little or no detectable protein in normal adult tissues [[24]] except in placentas, germinal centers in lymph nodes, and gonads [[25]]. Re-activation of IMP3 expression is observed in many human cancers, including the ovary, the endometrium, and the cervix, correlating with increased risk of metastases and decreased survival [[24],[26]–[30]]. Not only overexpressed in the invasive cancers, IMP3 has also been considered as a marker of preinvasive lesions in the cervix and the endometrium [[26],[28],[31]]. IMP3 has also been used as a prognostic marker for all ovarian cancer patients in our routine pathology practice, during which IMP3 overexpression was sometimes observed in normal-appearing tubal mucosa as well as in STIC cases. We defined positive IMP3 cytoplasmic staining in more than 10 tubal epithelial cells in a continuous fashion as an IMP3 signature. The finding of IMP3 signatures in benign-appearing tubal epithelia and the IMP3 overexpression in STIC and PSC cases prompted us to examine the following questions: 1) whether IMP3 expression signifies an early form of tubal serous neoplasia; 2) what the relationship is between IMP3 and tubal SCE in the process of pelvic serous carcinogenesis; 3) to what extent the changes are in the IMP3 expression in high-risk patients as well as patients with PSC; and 4) whether these changes are independent of the ageing process.

### Materials and methods

#### Case collection

A total of 316 consecutively identified surgical cases including salpingectomy specimens between 2006 and 2013 were identified from pathology files of University of Arizona Medical Center in Tucson, Arizona. The study was approved by the institutional review board. Cases were divided into three groups of patients: low-risk (n = 196), high-risk (n = 60), and patients with PSC (n = 60). Low-risk patients served as the control group and consisted of those patients post hysterectomies and salpingectomies performed for benign diseases (leiomyomata, endometriosis or uterine prolapse). Controls were further divided into age groups of 10 year intervals to determine normal distribution of IMP3 signatures. The main reason to include more control subjects for the study was to examine if age is related to the increased incidence of IMP3 signatures. High-risk patients were those with either *BRCA* mutations (n = 16), history of breast cancer (n = 32) or first degree family history of ovarian cancer (n = 12). Typically, these patients underwent prophylactic bilateral salpingo-oophorectomy. The median and mean interval between previous breast cancer and prophylactic bilateral salpingo-oophorectomy were 78 and 85 months, respectively. The 60 PSC patients represented FIGO stage 2 (n = 5), stage 3 (n = 51) and stage 4 (n = 4). By convention, the primary sites for these PSC cases included ovary (n = 50), fallopian tube (n = 4), and peritoneum (n = 6). Age of patients was matched among the three groups. For controls, two representative sections of the fallopian tube, one from ampulla (proximal) and the other from fimbria were submitted.

#### Tissue handling

For high-risk and PSC groups, the entire fallopian tube was submitted based on SEE-FIM protocol [[2],[32]]. Fallopian tube from low-risk control cases were processed by embedding all fimbriated ends similar to cancer patients with additional representative 2 cross sections of the ampulla as described elsewhere [[7]].

#### Morphologic analysis

The secretory and ciliated cells within the tubal mucosa were readily identifiable under light microscopy. SCEs and SCOUTs were identified under light microscopy and stained with PAX8 and tubulin when morphologic identification was uncertain as described previously [[15]]. STIC is a noninvasive carcinoma confined to the epithelial cells of tubal mucosae and characterized by significant cytologic atypia and/or atypical intraepithelial proliferation. The diagnosis of STIC and PSC was confirmed by at least 2 pathologist co-authors as described previously [[15],[21],[32],[33]].

#### Immunohistochemical analysis

IMP3 antibody (L523S) was provided by Dako Corporation (Seattle, WA), which was mouse monoclonal antibody specific for IMP3/KOC antigen. Immunohistochemical stains were performed on 5-μm formalin fixed paraffin embedded tissue sections from representative blocks using the purified mouse anti-IMP3 antibody and the standard avidin-biotin-complex technique as described previously [[28]-[30],[34]]. Representative sections of endometrial serous carcinoma served as positive controls for the IMP3 antibody [[28]]. Negative controls were performed by replacing the primary antibody with non-specific IgG. All slides were reviewed independently by 2 investigators (YW and WZ). The percentage of neoplastic cells and nonneoplastic tissues that showed dark brown cytoplasmic staining was recorded. The intensity of the IHC staining was recorded as absent, weak, moderate, or strong. IMP3 overexpression in STIC or PSC was defined as >10% of the stained cancer cells with strong intensity of the cytoplasmic staining. PAX8 has been considered as a mullerian epithelial marker identifying tubal secretory, but not ciliated cells, α-Tubulin has been used to mark cellular surface cilia [[7],[14]].

#### Data evaluation and statistical analysis

The presence of IMP3 signatures was defined by 10 or more tubal secretory cells stained positively and continuously in benign appearing tubal mucosa. The following parameters were calculated for the cases studied: 1) the number of IMP3 signatures and their distribution (fimbria vs ampulla) were calculated according to age intervals; 2) the comparison of IMP3 signatures in ampulla and fimbria within the fallopian tube; 3) the frequency of IMP3 signatures in cases and controls; 4) the frequency of IMP3 signatures in STIC in cases and controls after correcting for age; and 5) the IMP3 overexpression in STIC and PSC.

The data were analyzed by standard contingency table methods and nonparametric Mann–Whitney U-tests using the Eproliferative index LOG (Epicenter Software, Pasadena, CA, USA) and Stat View computer programs. To adjust age differences and the varying numbers of section or microscopic fields examined for each case, the data were calculated on the assumption that the number of IMP3 signatures in each case follows a Poisson distribution, which is commonly used to model count data, with an offset term used to account for the microscopic fields examined.

### Results

#### The incidence of IMP3 signatures increased with age

Cells with IMP3 signature represented tubal secretory cells. They were confirmed by both morphologic and immunohistochemcial stainings by PAX8 and tubulin as described previously [[15]]. There were a significantly increased number of IMP3 signatures in the fallopian tube with increasing age in all three groups (low-risk, high-risk, and PSC). This is consistent with our previous finding that secretory cells increase with age accompanied by decreased ciliated cells [[7]]. Within the low-risk group, the IMP3 signatures were not observed in patients under age 50, but the number of IMP3 signatures increased significantly in the fallopian tube in higher-age patients. Within the high-risk group, 5 IMP3 signatures appeared in the age group of 30–39, then the number of IMP3 signatures increased by approximately 20-fold in age group 70–79. A similar finding to IMP3 signature was found in the PSC group. Overall, the trend of increasing IMP3 signatures with age was statistically significant (*p* < 0.001) within all three groups.

**Figure 1:**
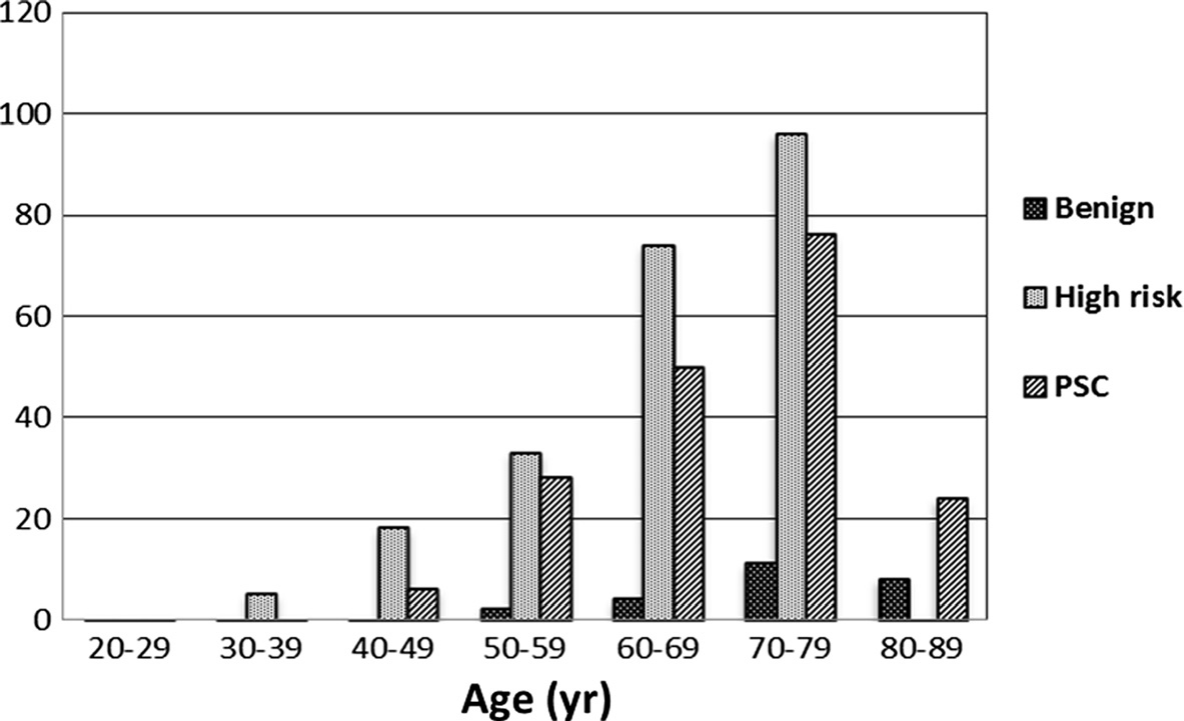
**The IMP3 signature increases with age.** The detailed data are presented in Table 1.

Regarding the tubal locations of IMP3 signatures, within the high-risk and PSC groups, the tubal fimbria had about 10-fold more IMP3 signatures than that in the ampulla region (p < 0.001). We observed significantly higher number of IMP3 signatures in the fimbria region from the patients of low-risk group. The detailed data about the IMP3 signatures in tubal segments are summarized in Table 1 and the corresponding bar graph is shown in Figure 1.

**Table 1:** Increased IMP3 signatures were independently associated with age, high-risk factors, and the status of pelvic serous carcinoma

**Age (Mean ± SD)**	**Low risk (n = 196)**		**High risk (n = 60) #IMP3-S/Group**		**PSC (n = 60)**	
	**fimbria**	**ampulla**	**fimbria**	**ampulla**	**fimbria**	**ampulla**
20–29 (25 ± 1.9)	0.00	0.00	0.00	0.00	-	-
30–39 (34 ± 1.8)	0.00	0.00	5	0.00	-	-
40–49 (46 ± 1.8)	0.00	0.00	18	0.00	6	0.00
50–59 (55 ± 1.6)	2	0.00	33	3	28	0.00
60–69 (63 ± 2.2)	4	0.00	74	8	50	3
70–79 (76 ± 1.4)	11	0.00	96	11	76	8
>80 (84 ± 2.3)	8	0.00	-	-	24	5
Total	25	0.00	226	22	184	16

PSC: pelvic serous carcinoma. #IMP3-S: the number of IMP3 signatures.

There was no high risk cases with age elder than 80 and no PSC cases younger than 40 in this study. There were only 5 PSC cases with age elder than 80.

#### IMP3 signatures were largely correlated with secretory cell expansions, but not with secretory cell outgrowth

**Figure 2:**
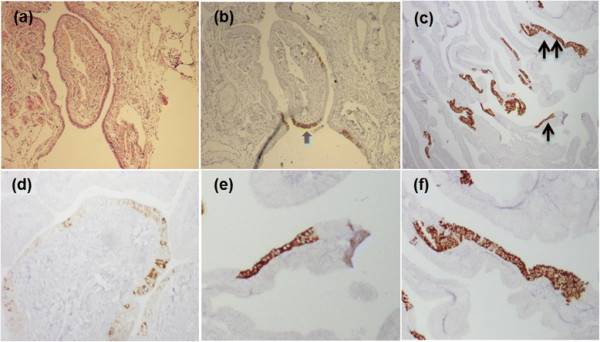
**IMP3 signatures in fallopian tube.** These tubal sections were derived from high-risk group **(a)**, **(b)**, PSC **(c)**, **(e)**, **(f)**, and low-risk group **(d)**. Panel **(b)** shows IMP3 signatures (arrow) in the low-mid area and the corresponding H&E staining shows morphologically unremarkable tubal epithelial cells **(a)**. Panel **(d)** shows sporadic cytoplasmic staining of IMP3 in a non-continuous pattern, which is not qualified as IMP3 signatures. Panel **(c)** shows multiple foci of IMP3 signatures with some corresponding to IMP3-SCE (single arrow, 10–30 positive cells in a row) and some corresponding to IMP3-SCOUTs (double arrow, ≥30 positive cells), which are magnified into panel **(e)** and panel **(f)**, respectively.

IMP3 positive tubal epithelial cells ranged from a couple of sporadic tubal epithelial cells to more than 30 epithelial cells in a row (Figure 2). Based on the positively IMP3-stained epithelial cells in tubal mucosal segment, we correlated them to the SCE and SCOUTs based on morphologic and immunophenotypic features of secretory and ciliated cells and the number of secretory cells in a row. After excluding focal staining without qualification of IMP3 signatures, we identified a total of 473 foci of IMP3 signatures in the 316 studied cases. Among them, the number of IMP3 signatures was present in 25 (average of 0.13), 248 (average of 4.13), and 200 (average of 3.33) foci per group cases in the low-risk, high-risk, and PSC group, respectively. This suggests that the number of IMP3 signatures per case was approximately 32 and 26 fold higher in high-risk and PSC group, correspondingly than those in the low-risk group (*p* < 0.000). Among the all IMP3 signatures, 388 (82%) corresponded to those morphologically identified SCE, termed as IMP3-SCE, while 85 (18%) represented SCOUTs, termed as IMP3-SCOUTs (*p* < 0.01). Representative pictures of IMP3 staining correlating to SCE and SCOUTs are presented in Figure 2. There were no single IMP3-SCOUTs present in the low-risk group; all of the 85 IMP3-SCOUTs foci were distributed in high-risk group (39, 46%) and PSC group (46, 54%) (*p* = 0.09).

#### The number of IMP3 signatures significantly increased in tubal fimbria of patients with high-risk or pelvic serous carcinoma

IMP3 signatures were compared between the case and control groups in an age matched fashion. The IMP3 signatures significantly increased in tubal segments in patients of high-risk and PSC groups. Compared with low-risk group, the number of IMP3 signatures in tubal segments dramatically increased with a total of 248 foci (9.9-fold increment) in high-risk group and a total of 200 microscopic foci (8-fold more) in PSC patients (*p* < 0.001). The IMP3 signatures were noted to be significantly higher in fimbria (226 foci) than in ampullae (22 foci) region (*p* < 0.001) in the high-risk group. However, IMP3 signatures observed in high-risk and PSC patients showed no statistically significant differences (Table 1).

#### IMP3 signature increment was independently associated with age, high-risk factors, and the status of pelvic serous carcinoma

Noting that the increased number of tubal IMP3 signatures is associated with age and more frequent in high-risk and PSC patients, we explored whether the increased number of IMP3 signatures in high-risk or PSC patients are independent of age. We addressed this question in a regression model that adjusted for age by linear regression analysis. The three groups (low-risk, high-risk, and PSC) of patients were divided according to 10-year intervals and average IMP3 signature frequencies for the intervals were compared. There were still significant differences in the increased IMP3 signatures between the case and control groups independent of the effects of increasing age (Table 2). When both the cases and controls were combined to provide a greater number for comparison, a significant correlation was observed with a determination coefficient of 0.153, which implies that only about 15% of the increase in IMP3 signatures could be attributed to age. Both the high-risk and PSC groups registered higher IMP3 signatures than low-risk controls (*p* < 0.001), with an average increase of 0.85 log counts for high-risk cases vs control and 0.87 log for PSC cases vs control. Therefore, IMP3 signatures are seen in increased frequency in advancing age, in patients with high-risk factors and in patients with known PSC.

**Table 2:** IMP3 signatures in cases and controls and IMP3 overexpression in serous tubal intraepithelial carcinoma and pelvic serous carcinoma

**Group**	**#case**	**Mean**	**IMP3 signatures**	**STIC**	**PSC**
		**age**	**(Total#)**	**#of + cases/HR (%)**	**#of + cases (%)**
LR	196	48.6	25	0 (0)	0 (0)
HR	60	46.2	226	5/9 (55)	0 (0)
PSC	60	61.5	184	11/19 (58)	38 (63)

HR: high-risk; STIC: serous tubal intraepithelial carcinoma; PSC: pelvic serous carcinoma. The frequency of IMP3 signatures was much higher in tubal fimbria of patients with high-risk (9.04-fold) and those with PSC (7.36-fold) than that in control group (*p* < 0.0001).

#### IMP3 signatures and serous tubal intraepithelial carcinoma were significantly more frequent in tubal segments of patients with high-risk or pelvic serous carcinoma

**Figure 3:**
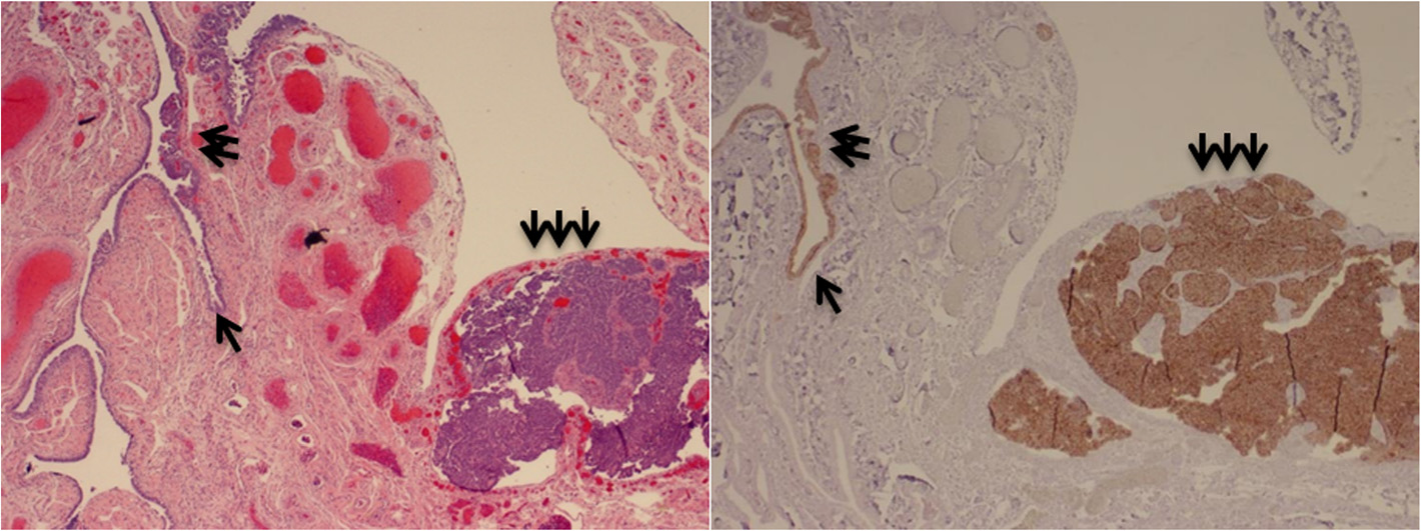
**IMP3 overexpression in tubal serous tubal intraepithelial carcinoma and invasive serous carcinoma.** IMP3 signatures (single arrow, right) shows morphologically bland cells (single arrow, left), which exists in anatomic continuity with tubal serous intraepithelial carcinoma (double arrows). The IMP3 is also diffusely positive in the invasive component of the serous carcinoma (triple arrows), which is located adjacent to the tubal fimbria in a case with PSC.

As previously defined, IMP3 signatures contain 10 or more secretory cells in a continuous fashion. IMP3 staining is most frequently observed in secretory cells since the majority of IMP3 signatures tubal segments showed homogeneous staining of a linear contiguous population of PAX8-positive cells (secretory cell marker) lacking the distinctive tubulin (ciliated cell marker) expression (data not shown). Considering the overall number of STIC is much less than IMP3 signatures, we calculated the frequency of positive cases regardless of the number of foci of lesions found in a single case. Overall, the frequency of IMP3-SCE (82%) was more prevalent than IMP3-SCOUTs (18%) in both high-risk and PSC groups. The absolute frequency of STIC in tubal fimbria in the three groups was 0% (0/196), 15% (9/60), and 32% (19/60). The STIC data are comparable to previous findings [[32],[33]]. Among the 9 STIC cases in high-risk group, strong cytoplasmic IMP3 staining (appearing in more than 50% of the neoplastic cells) was found in 5 (55%) cases; similarly, among the 19 STIC cases in PSC group, 11 (58%) were found with strong IMP3 staining. Furthermore, the positive IMP3 expression was found in 38 (63%) of 60 PSC cases. Interestingly, all 11 STIC cases in PSC group with positive IMP3 were also positive for IMP3 in the corresponding invasive cancer area; yet IMP3 signatures in these STIC cases were mainly located in the areas mostly adjacent to the areas of STIC. Representative pictures of IMP3 signatures and IMP3 overexpression in STIC as well as in PSC are illustrated in Figure 3.

### Discussion

In this study, we have examined IMP3 signature and its relationship with the aging process in patients with low-risk, high-risk, and PSC. For patients in the low-risk of PSC (control group), IMP3 signatures increased from 0 in patients <50 years of age to 11 in patients >70 years of age. The IMP3 signatures steadily increased with age, which is consistent to well-known epidemiologic findings that PSC increases with age and shows a peak incidence after menopause [[17],[35],[36]]. A more prominent increase of IMP3 signatures is observed in patients with high-risk factors, such as *BRCA* mutations or first degree family history of ovarian cancer, and in patients with PSC. Increased IMP3 signatures are also closely associated with age in both high-risk and PSC patients’ groups. Importantly, we found that age alone seems an independent risk factor, although it is less dramatic than the likely genetic alterations affecting high-risk and PSC populations, which have been well demonstrated in other studies [[2],[37],[38]]. As approximately 90% of women’s pelvic serous cancers including tubal, ovarian, and peritoneal origins are sporadic without identifiable genetic reasons [[39]], aging alone may play a role in serous neoplasia. Future studies addressing the connection between aging and pelvic serous neoplasia are needed to shed light on potential preventive strategies.

Tubal epithelia contain two distinctive cell types: secretory and ciliated cells. It is believed that tubal secretory cells represent the cell of origin for the majority PSCs in women. After we identified the phenomenon of IMP3 signatures in tubal epithelia, we further verified that IMP3 staining is also mainly localized in the tubal secretory cells rather than in the ciliated cells. The current model for the pelvic serous carcinogenesis starts from the expansion of secretory cells and the loss of tubal ciliated cells, which result in morphologically identifiable SCE and/or SCOUTs. These expanded segments of secretory cells are usually distributed evenly within the tubal fimbria and ampulla segments. In this study, however, we found that IMP3 signatures (tubal secretory cells positive for IMP3) are about 10-fold more frequent in tubal fimbria than that in the ampulla region (*p* < 0.001). Assuming all the secretory cells are equally susceptible to serous neoplasia, the frequency of serous STIC or early invasive carcinoma should be equally distributed in those tubal regions. However, many studies in the last decade revealed that majority STICs with or without early invasions are located in the tubal fimbria, while only a small percentage are in the tubal ampulla [[16],[40]–[42]]. This is supportive of previous observations that the fimbriae are the main site of origin for the serous neoplasia. It is currently unclear what the exact underlying molecular mechanism is for tubal fimbria serving as the most vulnerable segment to develop serous cancers. Apparently, regulatory factors of the cell cycle may be involved in this process. Alteration of *TP53* including p53 signatures has been recognized as an important initial stage for the development of PSC. Tubal epithelia with p53 signatures are favoured as latent precancer cells of PSC and they are also more frequently located in tubal fimbria [[5],[16]]. Our finding of IMP3 signatures being more prevalent in tubal fimbria may represent an additional important biomarker for serous cancer development. The relationship between the TP53 and IMP3 in the process of tubal serous carcinogenesis is under active study in our laboratory.

Both SCEs and SCOUTs have been considered as valid biomarkers for PSC since they are linked to serous neoplasia [[7],[8],[13],[15],[43]]. In this study, we showed that the frequency of IMP3-SCE is more common than IMP3-SCOUTs in all patients studied, which supports the recent findings that SCE is more sensitive than SCOUTs in association with serous neoplasia [[15]]. Since IMP3 staining is easily performed and the IMP3 signatures are readily identifiable under routine microscopy, it can serve as an additional biomarker in the process of serous carcinogenesis. Tubal secretory cells with IMP3 overexpression likely have growth advantage over secretory cells without IMP3 overexpression or ciliated cells. Such growth advantage may offer them a better opportunity to grow and evolve into PSC. However, the predictive value of IMP3 signatures for the PSC development needs to be further studied and validated prior to its clinical application.

In addition to the IMP3 overexpression in benign-appearing tubal epithelia, IMP3 is also overexpressed in 55% of STIC cases in high-risk patients, 58% of STIC cases in PSC and in 63% PSC cases. More interestingly, many IMP3 signatures were demonstrated to exist in anatomic continuity with STIC, suggesting a step-wise development from IMP3 signatures to STIC, then to PSC and further supporting that overexpression of IMP3 may be involved in the initial process of tubal or pelvic serous carcinogenesis.

## Abbreviations

PSC: Pelvic serous carcinoma

STIC: Serous tubal intraepithelial carcinoma

SCE: Secretory cell expansion

SCOUTs: Secretory cell outgrowths

## Competing interests

All the authors have no financial competing interest for the project.

## Authors’ contributions

YYW, ZJ, GY and WXZ conceived the study design and experiments. YYW, YW, WJZ, LL, and DL carried out experiments and data analysis. YYW, YW, WJZ, LL, DL, WXZ wrote the manuscript. All authors were involved in editing and approving the final manuscript.
